# MicroRNA Profiling of the Tears of Children With Vernal Keratoconjunctivitis

**DOI:** 10.3389/fgene.2022.847168

**Published:** 2022-04-12

**Authors:** Nazmul Huda Syed, Wan Nazatul Shima Shahidan, Ismail Shatriah, Embong Zunaina

**Affiliations:** ^1^ Department of Ophthalmology and Visual Science, School of Medical Sciences, Universiti Sains Malaysia, Kubang Kerian, Malaysia; ^2^ Basic Science and Oral Biology Unit, School of Dental Sciences, Universiti Sains Malaysia, Kubang Kerian, Malaysia; ^3^ Ophthalmology Clinic, Hospital USM, Kubang Kerian, Malaysia

**Keywords:** microRNA, microarrary, tears biomarkers, tears, vernal kerato conjunctivitis (VKC)

## Abstract

Vernal Keratoconjunctivitis (VKC) is a chronic conjunctival inflammatory condition that typically affects children. Extracellular microRNAs (miRNAs) are small noncoding RNA molecules, the expression of which is reported to regulate cellular processes implicated in several eye diseases. The aim of this preliminary study is to identify the miRNA expression profile in the tears of children with VKC *vis-à-vis* controls, and to statistically evaluate these miRNAs as potential diagnostic biomarkers of VKC. The study involved a VKC group and a control group. Tear specimens were collected using Schirmer’s strips. RNA was isolated using miRNeasy Micro kit and quantification was performed using an Agilent Bioanalyzer RNA 6000 Nano kit and Small RNA kit. miRNA profiling was performed using the Agilent microarray technique. A total of 51 miRNAs (48 upregulated and three downregulated) were differentially expressed in the tears of children with VKC and controls. The three most significantly upregulated miRNAs were hsa-miR-1229-5p, hsa-miR-6821-5p, and hsa-miR-6800-5p, and the three most significantly downregulated miRNAs were hsa-miR-7975, hsa-miR-7977, and hsa-miR-1260a. All the upregulated miRNAs are potential diagnostic biomarkers of VKC pending validation due to their larger discriminatory area under the curve (AUC) values. miRNA target prediction analysis revealed multiple overlapping genes that are known to play a role in conjunctival inflammation. We identified a set of differentially expressed miRNAs in the tears of children with VKC that may play a role in VKC pathogenesis. This study serves as the platform study for future miRNA studies that will provide a deeper understanding of the pathophysiology of VKC.

## Introduction

MicroRNAs (miRNAs) are small, 19–23 nucleotide-long noncoding RNA molecules that target messenger RNA (mRNA) to regulate gene expression (Simonson and Das, 2015). It has been posited that about 30% of human genes may be regulated by miRNAs (Lewis et al., 2005). Recent miRNA profiling studies on allergic diseases (e.g., bronchial asthma, eosinophilic esophagitis, allergic rhinitis, and atopic dermatitis) have identified specific miRNAs (i.e., miR-21, miR-146, miR-223, and miR-375) that play critical roles in regulating inflammatory mechanisms (Lu and Rothenberg, 2013). Studies using tears to investigate ocular diseases such as primary open-angle glaucoma, retinoblastoma, and dry eye syndrome have also reported the regulatory roles of miRNAs (Tamkovich et al., 2019; Lande et al., 2020; Wang et al., 2020).

Vernal keratoconjunctivitis (VKC) is an allergic inflammatory condition of the conjunctiva (Kumar, 2009). The symptoms include severe itching, constant tearing, photophobia, and mucus discharge, while the distinguishing characteristics of VKC include the presence of giant cobblestone papillae, conjunctival hyperemia, and trantas dots (Mathys and Lee, 2013). The age of onset of the disease is between 10 and 12 years old, with more young males affected than females (Bonini et al., 2000; Ukponmwan, 2003). The prevalence of VKC varies with geographical location and population, with being more common in temperate regions (Singhal et al., 2019).

The pathogenesis of VKC is multifactorial, complex, and not fully understood, and it involves numerous cellular signaling pathways that monitor the inflammatory cells in the conjunctiva of VKC patients (Mathys and Lee, 2013). Although often neglected, the genetics of an ocular allergy such as VKC is an important area that should be studied for better understanding of the disease. Though the symptoms and signs of VKC are clearly presented, VKC is a chronic and progressive form of allergic conjunctivitis which is often poorly managed. An early diagnosis of VKC would help in better management of the VKC and avoid severe damage to the cornea. Therefore, we aimed to explore the role of miRNAs, known as inflammatory regulators, in VKC for the first time. Tears contain extracellular miRNAs, which can potentially be used as informative diagnostic biomarkers to assess the pathophysiological condition of the ocular surface (Huang, 2017). Analyzing these extracellular miRNAs is challenging because of the particularly small sample volumes (less than 5 μL) of tears obtainable for study (von Thun und Hohenstein-Blaul et al., 2013). However, recent improvements in the detection sensitivities of profiling methods have facilitated the quantification of tear samples. Hence, this preliminary study uses a miRNA microarray to determine the differential miRNA expression in the tears of children with VKC *vis-à-vis* controls, and further, identifies and evaluates the miRNAs as potential diagnostic biomarkers for VKC.

## Materials and Methods

### Study Design and Subject Recruitment

A case-control study was conducted at the Hospital Universiti Sains Malaysia between February 2020 and January 2021. The study participants were children aged 6 to 17 years old who visited the hospital’s ophthalmology clinic. The study was conducted per the Declaration of Helsinki, and the study protocol was approved by the Research and Ethical Committee of the School of Medical Sciences, Universiti Sains Malaysia (USM/JEPeM/19090521). Written informed consent was obtained from the parents or legal guardians of all participants.

The participants—a total of eight children—were divided into two groups: a VKC group and a control group, with four children in each group. Inclusion criteria for the VKC group were being seven to 12 years old, a diagnosis, and active symptoms and signs of VKC. Children aged seven to 12 years old with no systemic or allergic diseases were recruited as the control group. Children with symptoms and signs of other ocular diseases or systemic allergic diseases, such as Steven Johnson Syndrome, bronchial asthma, rhinitis, and dermatitis, and those who were on systemic immunosuppressive therapy were excluded. The symptoms and signs of VKC were assessed and examined by a qualified consultant pediatric ophthalmologist.

### Tears Collection and Processing

Tear samples of the children with VKC and the controls were collected using Schirmers test strips. Schirmers strips were placed in the lower cu-de-sac region of each eye and subjects were instructed to close their eyes for 5 minutes, or at least, till the test strips were thoroughly wet to reduce discomfort. The soaked strips were carefully collected and immersed in phosphate buffer solution (PBS) within 1.5 ml tubes and stored at −80°C until further processing.

The absorbed Schirmer strips in 1.5 ml tubes within PBS were centrifuged at 6,000 g for 20 min at 4°C to remove debris. Following centrifugation, a clear supernatant was carefully transferred into fresh 1.5 ml tubes and stored at −80°C pending total RNA extraction. Total RNA extraction was performed using a miRNeasy micro kit (Qiagen, United States) per the manufacturer’s protocol, and its yield was quantified using a NanoDrop spectrophotometer (Thermo Fisher Scientific, United States). The quality of the total RNA and the presence of miRNAs were confirmed using an RNA Nano 6000 chip and a small RNA chip (Agilent Technologies, United States), respectively.

### Microarray Profiling

The RNA concentrations of all tear samples were normalized to 50 ng before performing a miRNA microarray analysis. A G3 Human miRNA Microarray Kit, 8 × 60 k (Agilent Technologies, United States) was used for miRNA profiling of the tear samples, per the manufacturer’s protocol. Raw expression data analysis was performed using the GeneSpring analysis software, version 14.9.1 (Agilent Technologies, United States).

### Target Genes Prediction

Target genes of differentially expressed miRNAs with a cut-off *p*-value < 0.05 and fold change (FC) of >2 were predicted using TargetScan database (Agarwal et al., 2015)^1^. Using AllerRGatlas, an allergic-related genes database, these target genes were screened for genes implicated in conjunctivitis (Liu et al., 2018).

### Statistics

Statistical analysis was performed using SPSS Statistics, version 26.0 (IBM, United States). The differential expression between the VKC group and the control group was determined using the student’s t-test, and multiple test correction was performed using the Benjamini–Hochberg multiple test correction procedure. The cut-off for significantly expressed miRNAs was set to a probability value of less than 5% (*p* < 0.05), and the FC was set to be greater than 2.0 (FC > 2). Total gene signal values were used to generate receiver operating characteristics (ROC) curves and area under the curve (AUC) scores to evaluate the differential miRNAs as potential diagnostic biomarkers of VKC based on their discriminatory scores.

## Results

### Tears Collection and Processing

The mean age of the VKC group was 9.75 years, while the mean age of the control group was 11.25 years. The various concentrations of the total RNA, small RNA, and miRNA are presented in Table 1. The total RNA concentrations measured using the NanoDrop spectrophotometer were lower than the readings obtained with the RNA Nano 6000 chip. Electropherograms of the RNA Nano 6000 chip readings show small RNA peaks less than 200 nucleotides in size, and the electropherograms of the readings of the small RNA chip reveal miRNA peaks less than 30 nucleotides in size (Supplementary Appendix S1). The bioanalyzer results show higher miRNA concentrations, despite disintegrated and low total RNA concentrations.

**TABLE 1:** Clinical characteristics and concentrations of total RNA and miRNA in tears among children with VKC and control groups.

Samples	Age	Gender	Familial history of allergy	Total RNA concentration (ng/μL)		miRNA concentration (ng/μL)
				Nanodrop	RNA nano 6000 chip	Small RNA chip
VKC1	9	M	Yes	62	8	2
VKC2	7	F	Yes	39	19	117
VKC3	12	M	Yes	22	15	7
VKC4	11	M	Yes	27	8	6
C1	10	M	No	24	6	3
C2	12	F	No	24	7	6
C3	11	F	No	29	13	6
C4	12	M	No	45	13	9

Abbreviation: VKC, Vernal keratoconjunctivitis; C, Control; miRNAs, MicroRNAs.

### MicroRNA Profiling

**FIGURE 1:**
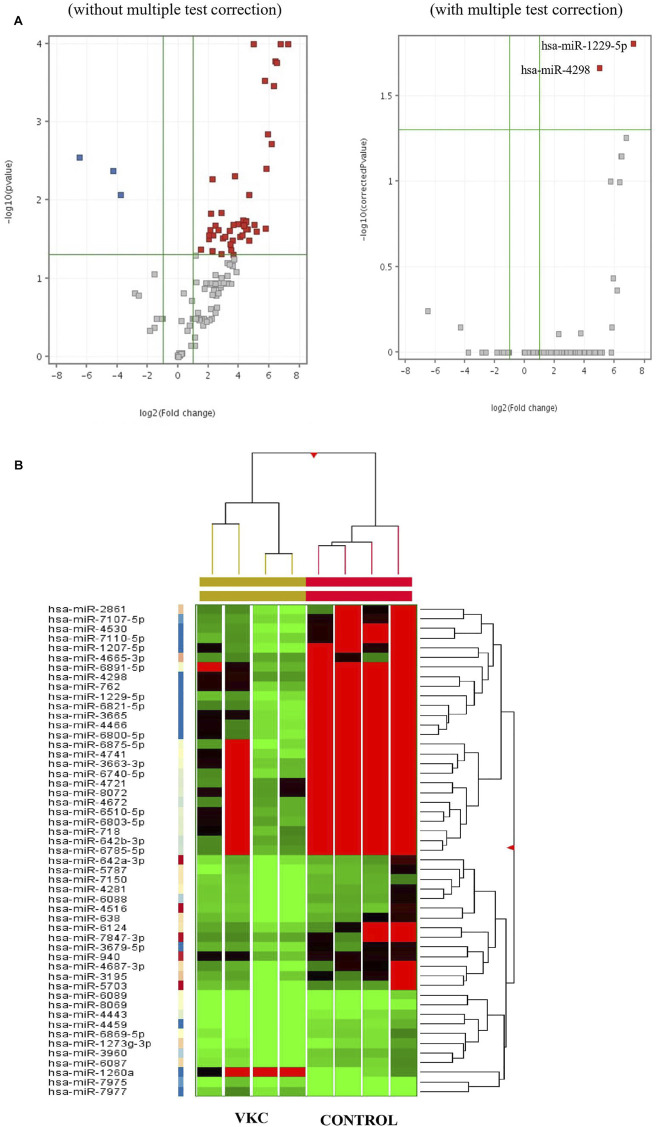
**(A)** Volcano plots visualizing the differentially expressed miRNAs in the tears of children with VKC and control both before and after multiple test correction. (Red: Entities which pass both corrected *p*-value and fold change cut-offs and are up-regulated, Dark Blue: Entities which pass both corrected *p*-value and fold change cut-offs and down-regulated, Grey: Entities which neither pass both corrected *p*-value and fold change cut-offs.). **(B)** Clustered heat map of all significantly expressed miRNAs in tears of children with VKC compared to control children. Moderated *t*-Test, *p* ≤ 0.05 and FC ≥ 2.0.

A total of 51 miRNAs were differentially expressed in the tears of children with VKC. Of the 51 miRNAs, 48 were significantly upregulated (Table 2A), while three miRNAs were significantly downregulated (Table 2B). The three most significantly upregulated miRNAs were hsa-miR-1229-5p, hsa-miR-6821-5p, and hsa-miR-6800-5p, while the three downregulated miRNAs were hsa-miR-7975, hsa-miR-7977, and hsa-miR-1260a. Only two miRNAs, hsa-miR-1229-5p and hsa-miR-4298, were reported after removing the false positive rates in the multiple test comparison using the Benjamini–Hochberg multiple test correction procedure. The miRNAs were filtered out and visualized *via* a volcano plot (Figure 1A). Hierarchical clustering analysis reveals a distinct expression of all differentially expressed miRNAs between the children with VKC and the controls (Figure 1B). Furthermore, on performing a review of the literature on each miRNA using an miRBase database (Griffiths-Jones et al., 2006), we observed that 24 of the 51 miRNAs have been reported to play a role in various types of cancers, including colorectal cancer (Kawaguchi et al., 2016), gastric cancer (Tsai et al., 2016), and hepatocellular carcinoma (Luo et al., 2016). The following miRNAs were found to be novel miRNAs that have not been yet linked to any pathological condition: hsa-miR-6800-5p, hsa-miR-7110-5p, hsa-miR-6740-5p, hsa-miR-4459, hsa-miR-8072, hsa-miR-7847-3p, hsa-miR-6869-5p, hsa-miR-6087, hsa-miR-8069, hsa-miR-1273g-3p, hsa-miR-7150, and hsa-miR-7975.

**TABLE 2:** **(A)** Significantly up-regulated miRNAs in tears of children with VKC in comparison to controls. **(B)** Significantly down-regulated miRNAs in tears of children with VKC in comparison to controls.

S.No	miRNA ID	*p*-Value	Fold change	*p*-Value after FDR testing	AUC value
1	hsa-miR-1229-5p	<0.001	7.25	0.016	1
2	hsa-miR-6821-5p	<0.001	6.74	NS	1
3	hsa-miR-6800-5p	<0.001	6.48	NS	1
4	hsa-miR-4466	<0.001	6.40	NS	1
5	hsa-miR-3665	<0.001	6.32	NS	1
6	hsa-miR-4530	<0.001	6.16	NS	1
7	hsa-miR-7110-5p	<0.001	5.91	NS	1
8	hsa-miR-1207-5p	<0.001	5.83	NS	1
9	hsa-miR-6875-5p	<0.001	5.77	NS	0.875
10	hsa-miR-762	<0.001	5.72	NS	1
11	hsa-miR-4741	0.003	5.17	NS	0.875
12	hsa-miR-6740-5p	0.002	5.02	NS	0.875
13	hsa-miR-4298	<0.001	4.99	0.022	1
14	hsa-miR-7107-5p	<0.001	4.70	NS	1
15	hsa-miR-2861	0.003	4.67	NS	1
16	hsa-miR-3663-3p	0.002	4.57	NS	0.875
17	hsa-miR-6891-5p	0.002	4.47	NS	0.875
18	hsa-miR-4672	0.002	4.47	NS	0.875
19	hsa-miR-6785-5p	0.002	4.47	NS	0.875
20	hsa-miR-6510-5p	0.002	4.36	NS	0.875
21	hsa-miR-6803-5p	0.021	4.35	NS	0.875
22	hsa-miR-718	0.021	4.31	NS	0.875
23	hsa-miR-642b-3p	0.018	4.29	NS	0.875
24	hsa-miR-6124	0.028	4.26	NS	1
25	hsa-miR-4687-3p	0.029	4.10	NS	1
26	hsa-miR-4721	0.020	3.96	NS	0.875
27	hsa-miR-4459	0.005	3.75	NS	1
28	hsa-miR-8072	0.021	3.66	NS	0.875
29	hsa-miR-5703	0.049	3.65	NS	1
30	hsa-miR-3195	0.033	3.58	NS	1
31	hsa-miR-7847-3p	0.044	3.50	NS	1
32	hsa-miR-4665-3p	0.036	3.47	NS	1
33	hsa-miR-6869-5p	0.025	3.38	NS	1
34	hsa-miR-638	0.029	3.12	NS	1
35	hsa-miR-6087	0.030	2.96	NS	1
36	hsa-miR-4516	0.049	2.88	NS	1
37	hsa-miR-3960	0.014	2.84	NS	1
38	hsa-miR-6089	0.024	2.66	NS	1
39	hsa-miR-4443	0.021	2.44	NS	1
40	hsa-miR-5787	0.028	2.31	NS	1
41	hsa-miR-642a-3p	0.045	2.25	NS	1
42	hsa-miR-3679-5p	0.005	2.25	NS	1
43	hsa-miR-6088	0.015	2.16	NS	1
44	hsa-miR-8069	0.024	2.14	NS	1
45	hsa-miR-4281	0.026	2.14	NS	1
46	hsa-miR-1273g-3p	0.031	2.01	NS	1
47	hsa-miR-7150	0.028	2.01	NS	1
48	hsa-miR-940	0.043	1.49	NS	1

S.No	miRNA ID	*p*-value	Fold change	*p*-Value after FDR testing	AUC value
1	hsa-miR-7975	0.0086	-13.63	NS	1
2	hsa-miR-7977	0.0042	-19.75	NS	1
3	hsa-miR-1260a	0.0028	-91.04	NS	0.625

Abbreviation: miRNA, microRNA; AUC, Area under curve; NS, Not significant.

### Identification of miRNAs as the Potential Biomarkers

ROC curves were used to examine the discriminatory power of the identified miRNAs (Supplementary Appendix S2). Of the 48 upregulated miRNAs (Table 2A), 35 miRNAs had an AUC score of 1, while the remaining 13 miRNAs (has-miR-6875-5p, hsa-miR-4741, hsa-miR-6740-5p, hsa-miR-3663-3p, hsa-miR-6891-5p, hsa-miR-4672, hsa-miR-6785-5p, hsa-miR-6510-5p, hsa-miR-6803-5p, hsa-miR-718, hsa-miR-642-3p, hsa-miR-4721, and hsa-miR-8072) had an AUC score of 0.875. Of the three downregulated miRNAs (Table 2B), hsa-miR-7975 and hsa-miR-7977 had an AUC score of 1, while hsa-miR-1260a had an AUC score of 0.625. These results show that all 48 upregulated miRNAs from the tears of children with VKC have better AUC scores (≥0.875) than the AUC scores of the downregulated miRNAs (≥0.625).

### Target Gene Prediction for Differentially Expressed miRNAs

A total of 2,290 target genes were acquired to determine the target genes of the 51 miRNAs. Target gene prediction was performed using the TargetScan database. Using the AlleRGatlas database, only 16 genes were found to be implicated in all kinds of conjunctivitis. The overlapping gene targets of the 13 most significantly upregulated miRNAs and all three downregulated miRNAs for VKC are presented in Table 3.

**TABLE 3:** Top 13 up-regulated and three down-regulated miRNAs in children with VKC in comparison to controls along with their conjunctivitis related gene targets.

No	miRNA ID	Total predicted target (TargetScan)	Inflammatory target genes
Up-regulated miRNAs			
1	hsa-miR-1229-5p	2,665	TGM2, MIF
2	hsa-miR-6821-5p	1,121	LGALS9
3	hsa-miR-6800-5p	2,469	B3GAT1
4	hsa-miR-4466	1,352	ICOSLG, PGF
5	hsa-miR-3665	4,650	ICOSLG, CCL22, CD276, NGFR, HRH1
6	hsa-miR-4530	5,517	ICOSLG, CCL22, LGALS9, CD276, NGFR, PGF
7	hsa-miR-7110-5p	4,250	PTGDS, ICOSLG, TGM2, CCL22, CD276, NGFR, FOXP3, HRH1, PGF
8	hsa-miR-1207-5p	6,300	PTGDS, ICOSLG, LGALS9, TGM2, MLF, CCL22, CD276, NGFR, FOXP3
9	hsa-miR-6875-5p	3,901	ARHGEF5, ICOSLG, B3GAT1, CCL22, CD276, FOXP3, NGFR
10	hsa-miR-762	5,621	PTGER1, B3GAT1, SOCS3, ICOSLG, TGM2, MMP25, NGFR, FOXP3, HRH1
11	hsa-miR-4741	4,565	NGFR, HRH1, FOXP3, PTGER1, SOCS3, MMP25
12	hsa-miR-6740-5p	3,798	FOXP3, SOCS3
13	hsa-miR-4298	3,401	LGALS9, ICOSLG, CCL22, HRH1, FOXP3, SOCS3
Down-regulated miRNAs			
11	hsa-miR-7975	2,907	NGFR
12	hsa-miR-7977	7,085	PTGDS, ARHGEF5, ICOSLG, B3GAT1, CCL22, NGFR, HRH1
13	hsa-miR-1260a	5,245	SOCS3, B3GAT1, NGFR, FOXP3, PGF

Predicted conjunctivitis-related genes retrieved from AllerGAtlas database are listed below: ARHGEF5, Rho guanine nucleotide exchange factor 5; CCL22, C-C motif chemokine ligand 22; CD276, CD276 molecule; LGALS9, galectin 9; MIF, macrophage migration inhibitory factor; PGF, placental growth factor; PTGDS, prostaglandin D2 synthase; PTGER, prostaglandin E receptor 1; B3GAT1, beta-1, 3-glucuronyltransferase 1; SOCS3, suppressor of cytokine signaling 3; ICOSLG, inducible T cell costimulator ligand; TGM2, transglutaminase 2; MMP25, matrix metallopeptidase 25; NGFR, nerve growth factor receptor; FOXP3, forkhead box P3; HRH1, histamine receptor H1.

## Discussion

MiRNAs are reported to regulate disease pathogenesis and maintain normal development and function (Alvarez-Garcia and Miska, 2005). Recent studies have linked miRNAs to ocular diseases such as Sjögren syndrome (Alevizos et al., 2011; Shi et al., 2014), trachoma (Derrick et al., 2013), cataract (Dunmire et al., 2013), myopia (Chen et al., 2012), retinoblastoma (Zhao et al., 2009), and pterygium (Engelsvold et al., 2013). Only a few studies have investigated the role of miRNAs in inflammatory ocular diseases such as allergic conjunctivitis in mice (Sun et al., 2015). Therefore, we report our observations on a new set of miRNAs expressed in children with VKC.

We observed a distinct total RNA yield and low RNA purity among the two study groups. It has previously been reported that tears contain diverse and high concentrations of miRNAs and are promising for biomarker discovery in ocular surface diseases (Weber et al., 2010). In contrast to the conventional use of exosomes, which some researchers claim are enriched sources of miRNAs (Zhao et al., 2017), we extracted miRNAs from unfractionated whole tears. Though the total RNA derived from the whole tears had lower total RNA concentrations, the bioanalyzer readings reveal substantially good concentrations of small RNA and miRNA. Lower purity levels of the total RNA were probably due to contaminants, such as proteins, phenol, and guanidine thiocyanate, absorbed at their respective wavelengths. Our findings parallel the results of previous studies, which have demonstrated that despite RNA degradation and lower total RNA purity levels, the miRNAs were stably expressed, and expression was not affected (Jung et al., 2010; Hall et al., 2012).

The microarray profiling reveals that out of 1,917 recorded human miRNAs in the miRBase database (Release 22.2) (Griffiths-Jones et al., 2006), a total of 51 miRNAs are differentially expressed in the tears of children with VKC *vis-à-vis* the controls in our study. This suggests that the expression of these 51 miRNAs may be associated with VKC. From our literature review, 24 miRNAs are implicated in various forms of cancer, while the other 12 miRNAs have no reported links to any published pathological condition. Such a large number of novel miRNAs reveals that the miRNA research on tears is far from exhaustively researched.

The remaining miRNAs have been reported to play roles in various systemic diseases. The miRNA, hsa-miR-638, is the most studied miRNA and is reported to play a role in colorectal cancer (Zhang et al., 2014), while hsa-miR-4530 is reported to be involved in regulating inflammatory response (Pagliari et al., 2017). Furthermore, hsa-miR-4672 has been reported to target genes related to the innate immune system (Kumari et al., 2016).

We found no previously reported miRNA studies on children with VKC. Therefore, we have tried to examine the relationships between their expression and other allergic inflammatory diseases. Liu et al. (2012) demonstrated that hsa-miR-1260a is significantly upregulated (FC = 2.2) in children with bronchial asthma. However, hsa-miR-1260a is significantly downregulated (FC = 6.5) in children with VKC. Furthermore, from the findings of this study, both hsa-miR-762 and hsa-miR-1207 were significantly upregulated in tears treated with the *Pseudomonas aeruginosa* antigen (Mun et al., 2013). It was further demonstrated that tear fluid regulates the innate defense mechanism by attuning epithelial miRNA expression. A study using the tears of patients with Sjögren syndrome revealed 14 miRNAs that are differentially expressed (Kim et al., 2019). However, none of these 14 miRNAs were expressed in the tears of children with VKC.

The ROC and AUC values were calculated and tabulated alongside the miRNAs as an additional step to examine the potential of these miRNAs for use as diagnostic biomarkers of VKC. Thirty-five upregulated miRNAs reveal an outstanding discrimination between the two groups with an AUC value of 1.0. The remaining 13 upregulated miRNAs had an AUC score in the range of 0.8–0.89, which is also incredibly discriminatory. Unfortunately, the AUC score of the downregulated miRNAs fell in the range of 0.6–1.0, which suggests that the diagnostic test has discriminatory ability (Mandrekar, 2010). Though the downregulated hsa-miR-1260a has the highest FC, of >−91, it recorded an unacceptably low AUC score, probably due to the very low sample size (Kamal et al., 2020). Thus, the two significantly upregulated miRNAs after multiple testing, hsa-miR-1229-5p and hsa-miR-4298, can better serve as potential diagnostic biomarkers for VKC. However, low sample size is a limitation of our study and validation of this work is planned with an appropriate number of participants in the future.

Target gene analysis of 51 miRNAs using TargetScan (Agarwal et al., 2015) revealed that a proline/arginine-rich end leucine-rich repeat protein (PRELP), also known as prolargin, is the most significant gene (*p*-value = 4.43E-08) targeting 35 differentially expressed miRNAs of VKC (Supplementary Appendix S3). PRELP has previously been reported in various body tissues, such as the skin, heart, sclera, and lung. Happonen et al. (2012) reported that *PRELP* inhibits all complement system pathways limiting the inflammatory response in rheumatoid arthritis. Thus, there is a need for further investigation of the role of PRELP in VKC. The other significant target genes were cyclin dependent kinase 5 regulatory subunit 2 (CDK5R2) and G protein-coupled receptor kinase 2 (GRK2), which are targeted by 25 and 21 miRNAs, respectively. *CDK5R2* is a pro-inflammatory gene reported in inflammatory bowel disease, while *GRK2* is a known immune cell regulator present in different types of immune cells (Han et al., 2018). The numerous predicted target genes reveal the potential biological pathways of interest involved in the pathogenesis of VKC. Furthermore, the other set of top three target genes targeted by miR-1229-5p and miR-4298 after multiple testing were oligosaccharyltransferase complex subunit 4 (OCT4), interleukin 17 receptor C (IL17RC), and pyrimidinergic receptor P2Y4 (P2RY4) (Supplementary Appendix S3). *IL17RC* partners with IL-17, which is a proinflammatory cytokine known to regulate inflammation (Hu et al., 2010). Therefore, we speculate further functional studies investigating the regulatory effects of these miRNAs would provide a deep understanding of VKC pathogenesis.

Notably, the miRNAs target a total of 16 overlapping genes (ARHGEF5, CCL22, CD276, LGALS9, MIF, PGF, PTGDS, PTGER1, B3GAT1, SOCS3, ICOSLG, TGM2, MMP25, NGFR, FOXP3, and HRH1), which are linked to all forms of conjunctivitis (Table 3). For instance, the placental growth factor (PGF) gene is associated with the NF-kappa family pathway, which is a major regulator of inflammatory responses (Liu et al., 2017). The suppressor of the cytokine signaling 3 (SOCS3) gene family is the negative regulator of the cytokine signaling pathway, and cytokines are reported to initiate and regulate immune responses (Oberholzer et al., 2000). A nerve growth factor receptor (NGFR) binds to a nerve growth factor (NGF). Bonini et al. (1996) postulated that NGF levels were elevated in patients with allergic inflammatory diseases and asthma. Forkhead Box P3 (FOXP3) is the transcriptional regulator critical to the development and inhibition of regulatory T-cells (Treg). Treg cells maintain immune system homeostasis by regulating other leukocytes (Lu et al., 2017). Similarly, miRNAs and their associated targets may perform important functions in the regulation of various immune responses that cause conjunctival inflammation in VKC.

The most significant retrieved GO terms were system development, anatomical structure development, and multicellular organism development. Thus, we surmise that the miRNAs and their associated target genes perform key functions in cellular processes such as cell development, cell apoptosis, cell proliferation, and inflammatory immune responses involved in VKC. Among the three most significant KEGG pathways, oxidative phosphorylation and glycolysis are the key players in the inflammatory process. Pro-inflammatory cells such as macrophages and lymphocytes draw their energy from these processes to mount an inflammatory response (Lee and Hüttemann, 2014; Soto-Heredero et al., 2020).

In conclusion, this study demonstrates that there is a total of 51 differentially expressed miRNAs in the tears of children with VKC. Of these miRNAs, two significant miRNAs after multiple testing, hsa-miR-1229-5p and hsa-miR-4298, can be used as potential diagnostic biomarkers for VKC upon further validation. The three most significant upregulated miRNAs were hsa-miR-1229-5p, hsa-miR-6821-5p, and hsa-miR-6800-5p, while the three most significant downregulated miRNAs were hsa-miR-7975, hsa-miR-7977, and hsa-miR-1260a. Various overlapping target genes, such as ARHGEF5, CCL22, CD276, LGALS9, MIF, PGF, PTGDS, PTGER1, B3GAT1, SOCS3, ICOSLG, TGM2, MMP25, NGFR, FOXP3, and HRH1, were predicted to regulate inflammatory immune responses in VKC *via* various pathways, including the NF-kappa pathway, the cytokine signaling pathway, and *via* Treg cells.

## Data Availability

The datasets for this article are not publicly available due to concerns regarding participant/patient anonymity. Requests to access the datasets should be directed to the corresponding author.

## Footnotes

1
http://www.targetscan.org/vert_71/.

## References

[B1] Agarwal V. Bell G. W. Nam J. W. Bartel D. P. (2015). Predicting Effective microRNA Target Sites in Mammalian mRNAs. Elife 4, e05005. 10.7554/eLife.05005 PMC4532895 26267216

[B2] Alevizos I. Alexander S. Turner R. J. Illei G. G. (2011). MicroRNA Expression Profiles as Biomarkers of Minor Salivary Gland Inflammation and Dysfunction in Sjögren's Syndrome. Arthritis Rheum. 63 (2), 535–544. 10.1002/art.30131 21280008 PMC3653295

[B3] Alvarez-Garcia I. Miska E. A. (2005). MicroRNA Functions in Animal Development and Human Disease. Development 132 (21), 4653–4662. 10.1242/dev.02073 16224045

[B4] Bonini S. Bonini S. Lambiase A. Marchi S. Pasqualetti P. Zuccaro O. (2000). Vernal Keratoconjunctivitis Revisited. Ophthalmology 107 (6), 1157–1163. 10.1016/s0161-6420(00)00092-0 10857837

[B5] Bonini S. Lambiase A. Bonini S. Angelucci F. Magrini L. Manni L. (1996). Circulating Nerve Growth Factor Levels Are Increased in Humans with Allergic Diseases and Asthma. Proc. Natl. Acad. Sci. U.S.A. 93 (20), 10955–10960. 10.1073/pnas.93.20.10955 8855290 PMC38265

[B6] Chen K.-C. Hsi E. Hu C.-Y. Chou W.-W. Liang C.-L. Juo S.-H. H. (2012). MicroRNA-328 May Influence Myopia Development by Mediating thePAX6Gene. Invest. Ophthalmol. Vis. Sci. 53 (6), 2732–2739. 10.1167/iovs.11-9272 22447870

[B7] Derrick T. Roberts C. h. Rajasekhar M. Burr S. E. Joof H. Makalo P. (2013). Conjunctival MicroRNA Expression in Inflammatory Trachomatous Scarring. Plos Negl. Trop. Dis. 7 (3), e2117. 10.1371/journal.pntd.0002117 23516655 PMC3597489

[B8] Dunmire J. J. Lagouros E. Bouhenni R. A. Jones M. Edward D. P. (2013). MicroRNA in Aqueous Humor from Patients with Cataract. Exp. Eye Res. 108, 68–71. 10.1016/j.exer.2012.10.016 23146683

[B9] Engelsvold D. H. Utheim T. P. Olstad O. K. Gonzalez P. Eidet J. R. Lyberg T. (2013). miRNA and mRNA Expression Profiling Identifies Members of the miR-200 Family as Potential Regulators of Epithelial-Mesenchymal Transition in Pterygium. Exp. Eye Res. 115, 189–198. 10.1016/j.exer.2013.07.003 23872359 PMC4278354

[B10] Griffiths-Jones S. Grocock R. J. Van Dongen S. Bateman A. Enright A. J. (2006). miRBase: microRNA Sequences, Targets and Gene Nomenclature. Nucleic Acids Res. 34 (Suppl. l_1), D140–D144. 10.1093/nar/gkj112 16381832 PMC1347474

[B11] Hall J. S. Taylor J. Valentine H. R. Irlam J. J. Eustace A. Hoskin P. J. (2012). Enhanced Stability of microRNA Expression Facilitates Classification of FFPE Tumour Samples Exhibiting Near Total mRNA Degradation. Br. J. Cancer 107 (4), 684–694. 10.1038/bjc.2012.294 22805332 PMC3419950

[B12] Han C. Li Y. Wang Y. Cui D. Luo T. Zhang Y. (2018). Development of Inflammatory Immune Response-Related Drugs Based on G Protein-Coupled Receptor Kinase 2. Cell Physiol Biochem 51 (2), 729–745. 10.1159/000495329 30463058

[B13] Happonen K. E. Fürst C. M. Saxne T. Heinegård D. Blom A. M. (2012). PRELP Protein Inhibits the Formation of the Complement Membrane Attack Complex. J. Biol. Chem. 287 (11), 8092–8100. 10.1074/jbc.m111.291476 22267731 PMC3318720

[B14] Hu Y. Ota N. Peng I. Refino C. J. Danilenko D. M. Caplazi P. (2010). IL-17RC Is Required for IL-17A- and IL-17F-Dependent Signaling and the Pathogenesis of Experimental Autoimmune Encephalomyelitis. J.I. 184 (8), 4307–4316. 10.4049/jimmunol.0903614 20231694

[B15] Huang W. (2017). MicroRNAs: Biomarkers, Diagnostics, and Therapeutics. Methods Mol. Biol. 1617, 57–67. 10.1007/978-1-4939-7046-9_4 28540676

[B16] Jung M. Schaefer A. Steiner I. Kempkensteffen C. Stephan C. Erbersdobler A. (2010). Robust microRNA Stability in Degraded RNA Preparations from Human Tissue and Cell Samples. Clin. Chem. 56 (6), 998–1006. 10.1373/clinchem.2009.141580 20378769

[B17] Kamal N. N. S. N. M. Awang R. A. R. Mohamad S. Shahidan W. N. S. (2020). Plasma-and Saliva Exosome Profile Reveals a Distinct MicroRNA Signature in Chronic Periodontitis. Front. Physiol. 11, 587381. 10.3389/fphys.2020.587381 33329037 PMC7733931

[B18] Kawaguchi T. Komatsu S. Ichikawa D. Tsujiura M. Takeshita H. Hirajima S. (2016). Circulating microRNAs: a Next-Generation Clinical Biomarker for Digestive System Cancers. Ijms 17 (9), 1459. 10.3390/ijms17091459 PMC5037738 27598137

[B19] Kim Y. J. Yeon Y. Lee W. J. Shin Y. U. Cho H. Sung Y.-K. (2019). Comparison of MicroRNA Expression in Tears of Normal Subjects and Sjögren Syndrome Patients. Invest. Ophthalmol. Vis. Sci. 60 (14), 4889–4895. 10.1167/iovs.19-27062 31752018

[B20] Kumar S. (2009). Vernal Keratoconjunctivitis: A Major Review. Acta Ophthalmol. 87 (2), 133–147. 10.1111/j.1755-3768.2008.01347.x 18786127

[B21] Kumari B. Jain P. Das S. Ghosal S. Hazra B. Trivedi A. C. (2016). Dynamic Changes in Global microRNAome and Transcriptome Reveal Complex miRNA-mRNA Regulated Host Response to Japanese Encephalitis Virus in Microglial Cells. Sci. Rep. 6 (1), 20263. 10.1038/srep20263 26838068 PMC4738309

[B22] Lande K. Gupta J. Ranjan R. Kiran M. Torres Solis L. F. Solís Herrera A. (2020). Exosomes: Insights from Retinoblastoma and Other Eye Cancers. Ijms 21 (19), 7055. 10.3390/ijms21197055 PMC7582726 32992741

[B23] Lee I. Hüttemann M. (2014). Energy Crisis: the Role of Oxidative Phosphorylation in Acute Inflammation and Sepsis. Biochim. Biophys. Acta (Bba) - Mol. Basis Dis. 1842 (9), 1579–1586. 10.1016/j.bbadis.2014.05.031 PMC4147665 24905734

[B24] Lewis B. P. Burge C. B. Bartel D. P. (2005). Conserved Seed Pairing, Often Flanked by Adenosines, Indicates that Thousands of Human Genes Are microRNA Targets. Cell 120, 15–20. 10.1016/j.cell.2004.12.035 15652477

[B25] Liu F. Qin H.-B. Xu B. Zhou H. Zhao D.-Y. (2012). Profiling of miRNAs in Pediatric Asthma: Upregulation of miRNA-221 and miRNA-485-3p. Mol. Med. Rep. 6 (5), 1178–1182. 10.3892/mmr.2012.1030 22895815

[B26] Liu J. Liu Y. Wang D. He M. Diao L. Liu Z. (2018). AllerGAtlas 1.0: a Human Allergy-Related Genes Database. Database 2018, bay010. 10.1093/database/bay010 PMC5824776 29688358

[B27] Liu T. Zhang L. Joo D. Sun S. C. (2017). NF-κB Signaling in Inflammation. Signal. Transduct. Target. Ther. 2 (1), 1–9. 10.1038/sigtrans.2017.23 PMC5661633 29158945

[B28] Lu L. Barbi J. Pan F. (2017). The Regulation of Immune Tolerance by FOXP3. Nat. Rev. Immunol. 17 (11), 703–717. 10.1038/nri.2017.75 28757603 PMC5793224

[B29] Lu T. X. Rothenberg M. E. (2013). Diagnostic, Functional, and Therapeutic Roles of microRNA in Allergic Diseases. J. Allergy Clin. Immunol. 132 (1), 3–13. 10.1016/j.jaci.2013.04.039 23735656 PMC3737592

[B30] Luo Y. Wen X. Wang L. Gao J. Wang Z. Zhang C. (2016). Identification of microRNAs Involved in Growth Arrest and Apoptosis in Hydrogen Peroxide-Treated Human Hepatocellular Carcinoma Cell Line HepG2. Oxid. Med. Cel. Longev. 2016, 7530853. 10.1155/2016/7530853 PMC5002491 27597883

[B31] Mandrekar J. N. (2010). Receiver Operating Characteristic Curve in Diagnostic Test Assessment. J. Thorac. Oncol. 5 (9), 1315–1316. 10.1097/jto.0b013e3181ec173d 20736804

[B32] Mathys K. C. Lee W. B. (2013). “Vernal Keratoconjunctivitis,” in Ocular Surface Disease: Cornea, Conjunctiva and Tear Film (Philadelphia: Expert Consult-Online and Print (Elsevier Health Sciences)), 97–102. 10.1016/b978-1-4557-2876-3.00014-6

[B33] Mun J. Tam C. Chan G. Kim J. H. Evans D. Fleiszig S. (2013). MicroRNA-762 Is Upregulated in Human Corneal Epithelial Cells in Response to Tear Fluid and *Pseudomonas aeruginosa* Antigens and Negatively Regulates the Expression of Host Defense Genes Encoding RNase7 and ST2. PLoS One 8 (2), e57850. 10.1371/journal.pone.0057850 23469087 PMC3585208

[B34] Oberholzer A. Oberholzer C. Moldawer L. L. (2000). Cytokine Signaling-Rregulation of the Immune Response in normal and Critically Ill States. Crit. Care Med. 28 (4), N3–N12. 10.1097/00003246-200004001-00002 10807312

[B35] Pagliari M. Munari F. Toffoletto M. Lonardi S. Chemello F. Codolo G. (2017). *Helicobacter pylori* Affects the Antigen Presentation Activity of Macrophages Modulating the Expression of the Immune Receptor CD300E through miR-4270. Front. Immunol. 8, 1288. 10.3389/fimmu.2017.01288 29085364 PMC5649134

[B36] Shi H. Zheng L.-y. Zhang P. Yuqi C.-q. (2014). miR-146a and miR-155 Expression in PBMCs from Patients with Sjögren's Syndrome. J. Oral Pathol. Med. 43 (10), 792–797. 10.1111/jop.12187 24931100

[B37] Simonson B. Das S. (2015). MicroRNA Therapeutics: the Next Magic Bullet? Mrmc 15, 467–474. 10.2174/1389557515666150324123208 PMC4410078 25807941

[B38] Singhal D. Sahay P. Maharana P. K. Raj N. Sharma N. Titiyal J. S. (2019). Vernal Keratoconjunctivitis. Surv. Ophthalmol. 64 (3), 289–311. 10.1016/j.survophthal.2018.12.001 30550738

[B39] Soto‐Heredero G. Gomez de las Heras M. M. Gabandé‐Rodríguez E. Oller J. Mittelbrunn M. (2020). Glycolysis–a Key Player in the Inflammatory Response. FEBS J. 287 (16), 3350–3369. 10.1111/febs.15327 32255251 PMC7496292

[B40] Sun W. Sheng Y. Chen J. Xu D. Gu Y. (2015). Down-regulation of miR-146a Expression Induces Allergic Conjunctivitis in Mice by Increasing TSLP Level. Med. Sci. Monit. 21, 2000–2007. 10.12659/MSM.894563 26166175 PMC4509417

[B41] Tamkovich S. Grigor'eva A. Eremina A. Tupikin A. Kabilov M. Chernykh V. (2019). What Information Can Be Obtained from the Tears of a Patient with Primary Open Angle Glaucoma? Clinica Chim. Acta 495, 529–537. 10.1016/j.cca.2019.05.028 31153869

[B42] Tsai M.-M. Wang C.-S. Tsai C.-Y. Huang H.-W. Chi H.-C. Lin Y.-H. (2016). Potential Diagnostic, Prognostic and Therapeutic Targets of microRNAs in Human Gastric Cancer. Ijms 17 (6), 945. 10.3390/ijms17060945 PMC4926478 27322246

[B43] Ukponmwan C. U. (2003). Vernal Keratoconjunctivitis in Nigerians: 109 Consecutive Cases. Trop. Doct. 33 (4), 242–245. 10.1177/004947550303300419 14620434

[B44] von Thun und Hohenstein-Blaul N. Funke S. Grus F. H. (2013). Tears as a Source of Biomarkers for Ocular and Systemic Diseases. Exp. Eye Res. 117, 126–137. 10.1016/j.exer.2013.07.015 23880526

[B45] Wang Q. Xie X. Li H. Hao S. (2020). Discovery of microRNA Expression Profiles Involved in Regulating TGF-Β2 Expression in the Tears of Dry Eye Patients. Ann. Clin. Biochem. 57 (6), 420–428. 10.1177/0004563220961746 32936670

[B46] Weber J. A. Baxter D. H. Zhang S. Huang D. Y. How Huang K. Jen Lee M. (2010). The microRNA Spectrum in 12 Body Fluids. Clin. Chem. 56 (11), 1733–1741. 10.1373/clinchem.2010.147405 20847327 PMC4846276

[B47] Zhang J. Fei B. Wang Q. Song M. Yin Y. Zhang B. (2014). MicroRNA-638 Inhibits Cell Proliferation, Invasion and Regulates Cell Cycle by Targeting Tetraspanin 1 in Human Colorectal Carcinoma. Oncotarget 5 (23), 12083–12096. 10.18632/oncotarget.2499 25301729 PMC4322991

[B48] Zhao J.-J. Yang J. Lin J. Yao N. Zhu Y. Zheng J. (2009). Identification of miRNAs Associated with Tumorigenesis of Retinoblastoma by miRNA Microarray Analysis. Childs Nerv. Syst. 25 (1), 13–20. 10.1007/s00381-008-0701-x 18818933

[B49] Zhao L. Yu J. Wang J. Li H. Che J. Cao B. (2017). Isolation and Identification of miRNAs in Exosomes Derived from Serum of colon Cancer Patients. J. Cancer 8 (7), 1145–1152. 10.7150/jca.18026 28607588 PMC5463428

