# Supplementary material for: MicroRNA Profiling of the Tears of Children With Vernal Keratoconjunctivitis
Source: Front Genet. 2022 Apr 12;13:847168. doi: 10.3389/fgene.2022.847168 (PMC9039132; doi:10.3389/fgene.2022.847168)
Supplement: Supplementary file 1 [file DataSheet2.PDF]

S2 Appendix:

Receiver operating characteristic (ROC) curves

1. ROC curves of all 48 significantly up-regulated miRNAs in tears of children with VKC vis-à-vis healthy control:

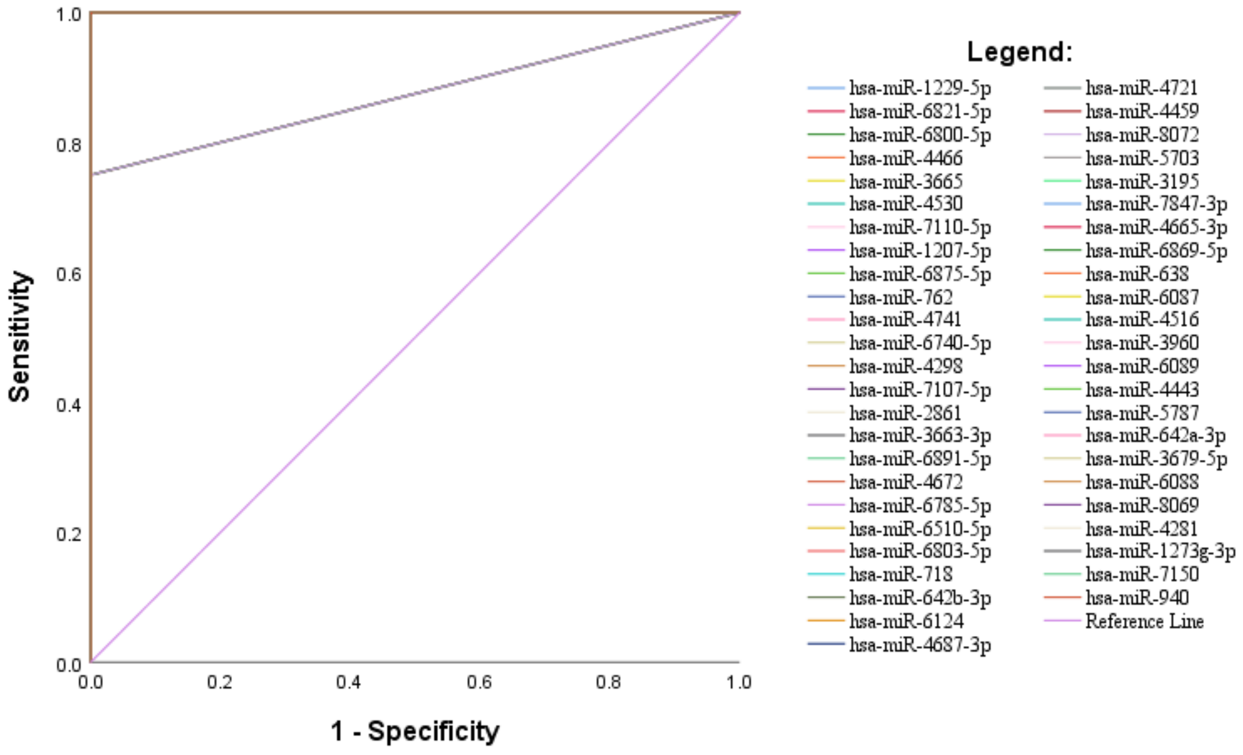

Area Under the Curve

Test Result Variable(s)	Area	Std. Error[a]	Asymptotic Sig.[b]	Asymptotic 95% Confidence Interval	
				Lower Bound	Upper Bound
hsa-miR-1229-5p	1.000	.000	.021	1.000	1.000
hsa-miR-6821-5p	1.000	.000	.021	1.000	1.000
hsa-miR-6800-5p	1.000	.000	.021	1.000	1.000
hsa-miR-4466	1.000	.000	.021	1.000	1.000
hsa-miR-3665	1.000	.000	.021	1.000	1.000
hsa-miR-4530	1.000	.000	.021	1.000	1.000
hsa-miR-7110-5p	1.000	.000	.021	1.000	1.000
hsa-miR-1207-5p	1.000	.000	.021	1.000	1.000
hsa-miR-6875-5p	.875	.143	.083	.594	1.000
hsa-miR-762	1.000	.000	.021	1.000	1.000
hsa-miR-4741	.875	.143	.083	.594	1.000
hsa-miR-6740-5p	.875	.143	.083	.594	1.000
hsa-miR-4298	1.000	.000	.021	1.000	1.000
hsa-miR-7107-5p	1.000	.000	.021	1.000	1.000
hsa-miR-2861	1.000	.000	.021	1.000	1.000
hsa-miR-3663-3p	.875	.143	.083	.594	1.000
hsa-miR-6891-5p	.875	.143	.083	.594	1.000
hsa-miR-4672	.875	.143	.083	.594	1.000
hsa-miR-6785-5p	.875	.143	.083	.594	1.000
hsa-miR-6510-5p	.875	.143	.083	.594	1.000
hsa-miR-6803-5p	.875	.143	.083	.594	1.000
hsa-miR-718	.875	.143	.083	.594	1.000
hsa-miR-642b-3p	.875	.143	.083	.594	1.000
hsa-miR-6124	1.000	.000	.021	1.000	1.000
hsa-miR-4687-3p	1.000	.000	.021	1.000	1.000
hsa-miR-4721	.875	.143	.083	.594	1.000
hsa-miR-4459	1.000	.000	.021	1.000	1.000
hsa-miR-8072	.875	.143	.083	.594	1.000
hsa-miR-5703	1.000	.000	.021	1.000	1.000
hsa-miR-3195	1.000	.000	.021	1.000	1.000
hsa-miR-7847-3p	1.000	.000	.021	1.000	1.000
hsa-miR-4665-3p	1.000	.000	.021	1.000	1.000

hsa-miR-6869-5p	1.000	.000	.021	1.000	1.000
hsa-miR-638	1.000	.000	.021	1.000	1.000
hsa-miR-6087	1.000	.000	.021	1.000	1.000
hsa-miR-4516	1.000	.000	.021	1.000	1.000
hsa-miR-3960	1.000	.000	.021	1.000	1.000
hsa-miR-6089	1.000	.000	.021	1.000	1.000
hsa-miR-4443	1.000	.000	.021	1.000	1.000
hsa-miR-5787	1.000	.000	.021	1.000	1.000
hsa-miR-642a-3p	1.000	.000	.021	1.000	1.000
hsa-miR-3679-5p	1.000	.000	.021	1.000	1.000
hsa-miR-6088	1.000	.000	.021	1.000	1.000
hsa-miR-8069	1.000	.000	.021	1.000	1.000
hsa-miR-4281	1.000	.000	.021	1.000	1.000
hsa-miR-1273g-3p	1.000	.000	.021	1.000	1.000
hsa-miR-7150	1.000	.000	.021	1.000	1.000
hsa-miR-940	1.000	.000	.021	1.000	1.000

The test result variable(s): Predicted probability, Predicted probability, Predicted probability, Predicted probability, Predicted probability, Predicted probability, Predicted probability, Predicted probability, Predicted probability, Predicted probability, Predicted probability, Predicted probability, Predicted probability has at least one tie between the positive actual state group and the negative actual state group. Statistics may be biased.

a. Under the nonparametric assumption
b. Null hypothesis: true area = 0.5

2. ROC curves of the three most significantly downregulated miRNAs in the tears of children with VKC vis-à-vis the control group:

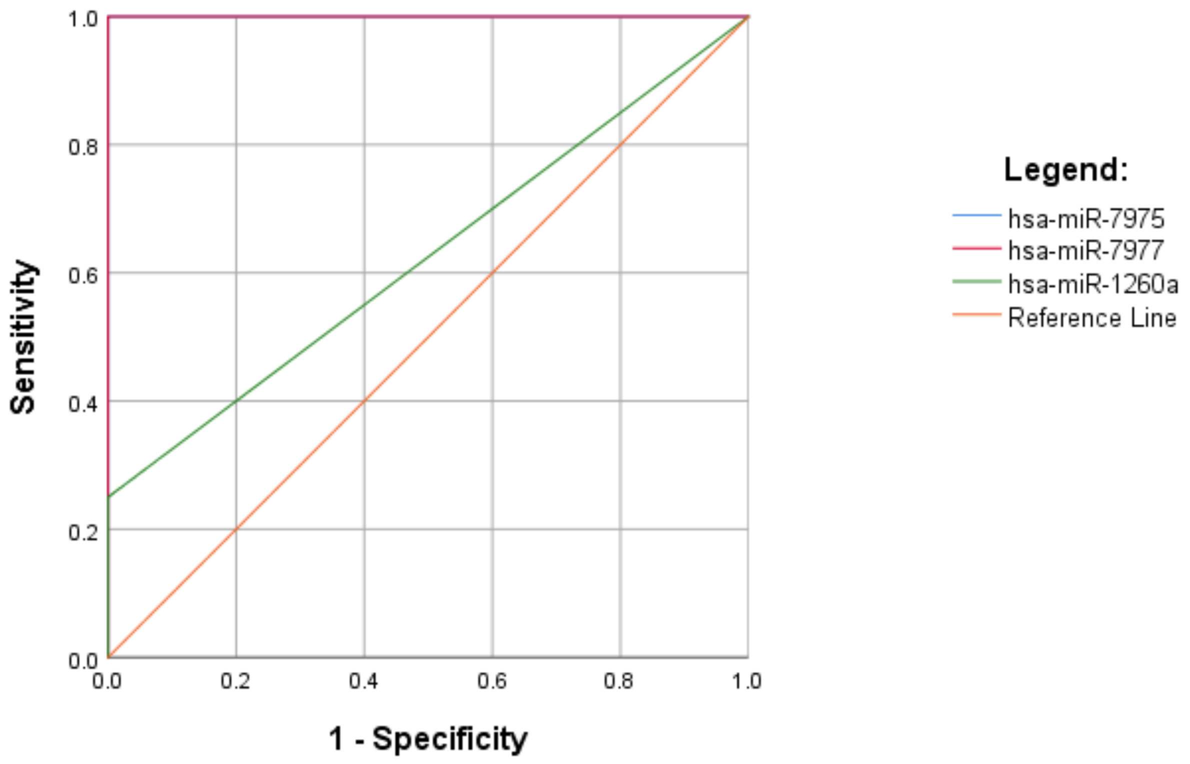

Area Under the Curve

Test Result Variable(s)	Area	Std. Error[a]	Asymptotic Sig.[b]	Asymptotic 95% Confidence Interval	
				Lower Bound	Upper Bound
Predicted probability	1.000	.000	.021	1.000	1.000
Predicted probability	1.000	.000	.021	1.000	1.000
Predicted probability	.625	.210	.564	.214	1.000

The test result variable(s): Predicted probability has at least one tie between the positive actual state group and the negative actual state group. Statistics may be biased.

a. Under the nonparametric assumption

b. Null hypothesis: true area = 0.5
