# Supplementary material for: MicroRNA Profiling of the Tears of Children With Vernal Keratoconjunctivitis
Source: Front Genet. 2022 Apr 12;13:847168. doi: 10.3389/fgene.2022.847168 (PMC9039132; doi:10.3389/fgene.2022.847168)
Supplement: Supplementary file 2 [file DataSheet3.PDF]

S3 Appendix: List of miRNA target genes using TargetScan database

(1) Top 10 target genes of all 51 miRNAs based on their P-values.

S.No	Gene symbol	Gene name	P-value
1	PRELP	proline and arginine rich end leucine rich repeat protein	4.43E-08
2	CDK5R2	cyclin dependent kinase 5 regulatory subunit 2	6.32E-08
3	GRK2	G protein-coupled receptor kinase 2	7.37E-08
4	REEP6	receptor accessory protein 6	7.83E-08
5	PLVAP	plasmalemma vesicle associated protein	2.34E-07
6	ZDHHC8	zinc finger DHHC-type containing 8	3.72E-07
7	HCAR1	hydroxycarboxylic acid receptor 1	5.29E-07
8	ADAP1	ArfGAP with dual PH domains 1	9.55E-07
9	NAT16	N-acetyltransferase 16 (putative)	1.57E-06
10	SUFU	SUFU negative regulator of hedgehog signaling	1.73E-06

(2) Top 10 target genes of two significant miRNAs after multiple testing based on their P-values.

S.No	Gene symbol	Gene name	P-value
1	OST4	oligosaccharyltransferase complex subunit 4, non-catalytic	0.002
2	IL17RC	interleukin 17 receptor C	0.003
3	P2RY4	pyrimidinergic receptor P2Y4	0.004
4	ACTL8	actin like 8	0.004
5	PHYKPL	5-phosphohydroxy-L-lysine phospho-lyase	0.005
6	GSTM1	glutathione S-transferase mu 1	0.006
7	CAPN3	calpain 3	0.007
8	LUZP4	leucine zipper protein 4	0.009
9	ABTB1	ankyrin repeat and BTB domain containing 1	0.009
10	CATSPERD	cation channel sperm associated auxiliary subunit delta	0.009
