# Supplementary material for: MicroRNA Profiling of the Tears of Children With Vernal Keratoconjunctivitis
Source: Front Genet. 2022 Apr 12;13:847168. doi: 10.3389/fgene.2022.847168 (PMC9039132; doi:10.3389/fgene.2022.847168)

S1 Appendix (1): Electropherograms of RNA Nano 6000 Chip showing the RNA Integrity Number (RIN) small RNA peaks (size < 200 nucleotides) between VKC and control groups.

VKC GROUP

CONTROL GROUP

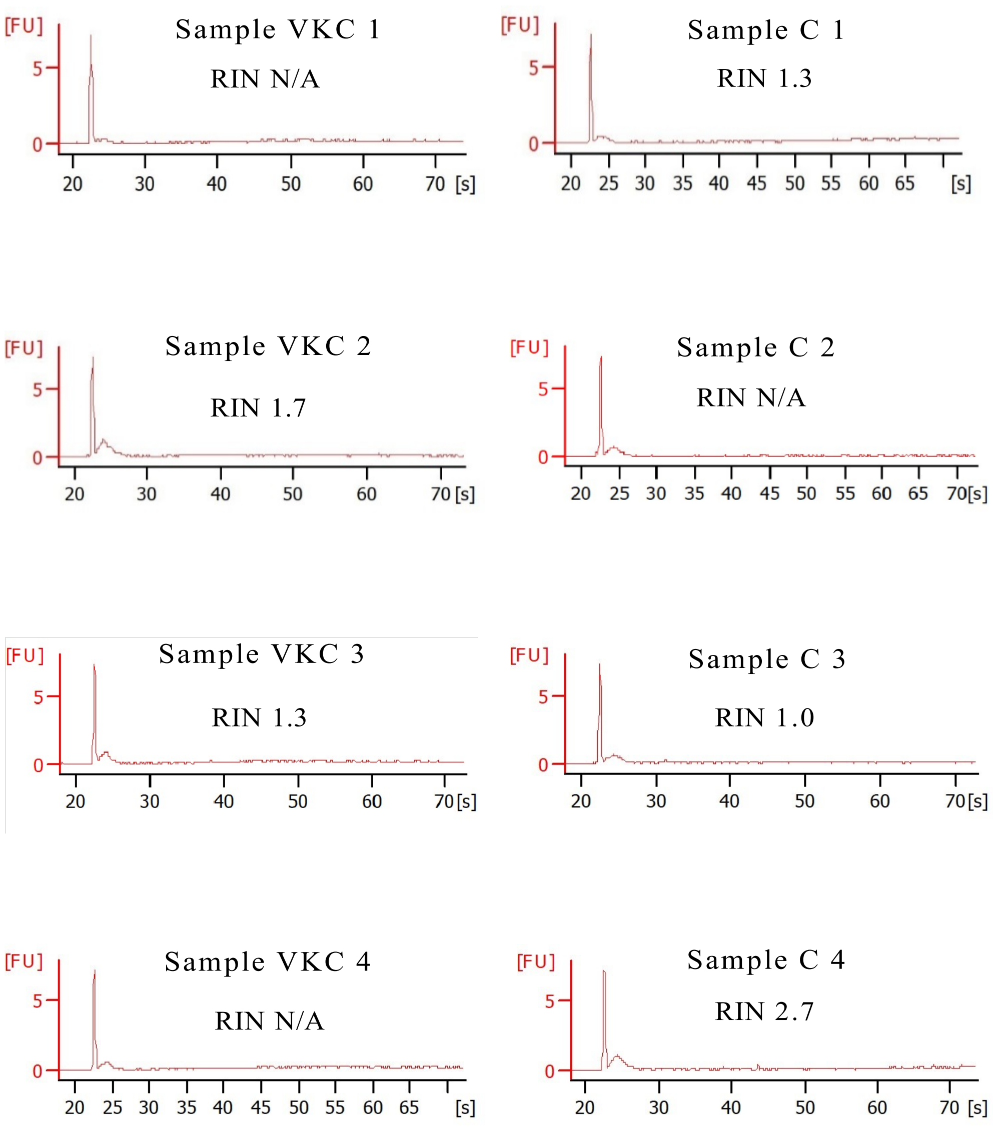

S1 Appendix (2): Electropherograms of Small RNA Chip using showing miRNA percentage and miRNA peaks (size < 30 nucleotides) between VKC and control groups.

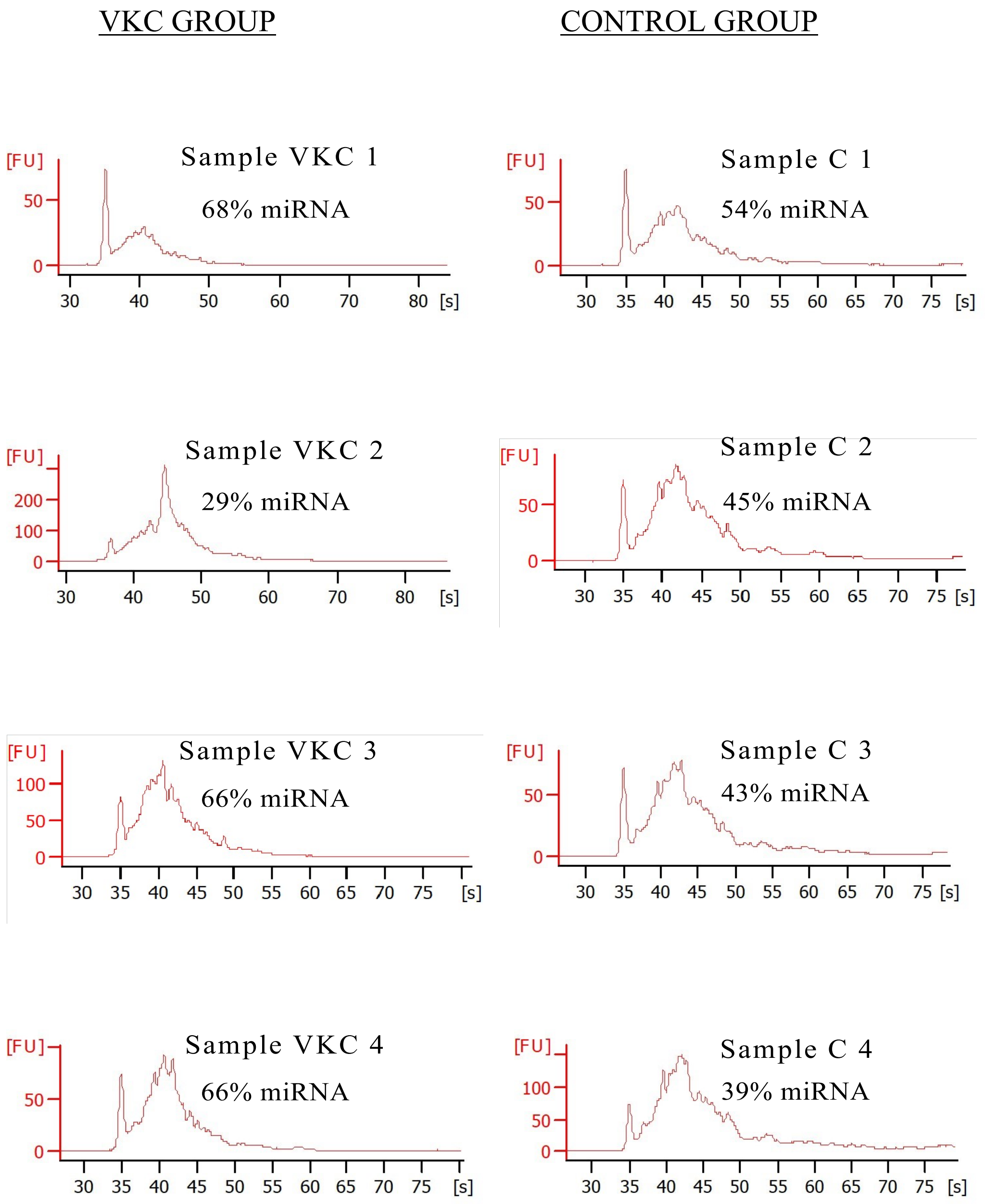

Supplement: Supplementary file 3 [file DataSheet1.PDF]
